# Hypertensive Disorders of Pregnancy and Offspring Cardiac Structure and Function in Adolescence

**DOI:** 10.1161/JAHA.116.003906

**Published:** 2016-10-31

**Authors:** Simon Timpka, Corrie Macdonald‐Wallis, Alun D. Hughes, Nishi Chaturvedi, Paul W. Franks, Debbie A. Lawlor, Abigail Fraser

**Affiliations:** ^1^ Genetic and Molecular Epidemiology UnitLund University Diabetes CenterLund UniversityMalmöSweden; ^2^ School of Social and Community MedicineUniversity of BristolUnited Kingdom; ^3^ MRC Integrative Epidemiology at the University of BristolUniversity of BristolUnited Kingdom; ^4^ Institute of Cardiovascular ScienceUniversity College LondonLondonUnited Kingdom; ^5^ Harvard T.H Chan School of Public HealthHarvard UniversityBostonMA

**Keywords:** ALSPAC, blood pressure, cohort study, concentric remodeling, echocardiography, epidemiology, hypertension, preeclampsia/pregnancy, Epidemiology, Primary Prevention, Pediatrics, Risk Factors, Remodeling

## Abstract

**Background:**

Fetal exposure to preeclampsia is associated with higher blood pressure and later risk of stroke. We aimed to investigate the associations of maternal preeclampsia, gestational hypertension, and maternal blood pressure change in pregnancy with offspring cardiac structure and function in adolescence.

**Methods and Results:**

Using data from a prospective birth cohort study, we included offspring who underwent echocardiography (mean age, 17.7 years; SD, 0.3; N=1592). We examined whether hypertensive disorders of pregnancy were associated with offspring cardiac structure and systolic/diastolic function using linear regression. Using multilevel linear spline models (measurement occasions within women), we also investigated whether rate of maternal systolic/diastolic blood pressure change during pregnancy (weeks 8–18, 18–30, 30–36, and 36 or more) were associated with offspring outcomes. Main models were typically adjusted for maternal age, offspring age and sex, prepregnancy body mass index, parity, glycosuria/diabetes mellitus, education, and maternal smoking. Exposure to maternal preeclampsia (0.025; 95% CI, 0.008–0.043) and gestational hypertension (0.010; 0.002–0.017) were associated with greater relative wall thickness. Furthermore, preeclampsia was also associated with a smaller left ventricular end‐diastolic volume (−9.0 mL; −15 to −3.1). No associations were found between hypertensive disorders of pregnancy and offspring cardiac function. Positive rate of maternal systolic blood pressure change during weeks 8 to 18 was associated with greater offspring left ventricular end‐diastolic volume, left ventricular mass indexed to height^2.7^, and E/A.

**Conclusions:**

Adolescent offspring exposed to maternal preeclampsia had greater relative wall thickness and reduced left ventricular end‐diastolic volume, which could be early signs of concentric remodeling and affect future cardiac function as well as risk of cardiovascular disease.

## Introduction

Preeclampsia is diagnosed in 2% to 5% of pregnancies, with the incidence for other forms of hypertension in pregnancy being about twice as high.^1^ Whereas “gestational hypertension” describes new hypertension that manifests in previously normotensive women following the 20th week of pregnancy, in preeclampsia the hypertension is typically complicated by concomitant proteinuria. A complex syndrome of suboptimal placentation, increased levels of antiangiogenic factors, and fetal hypoxia, preeclampsia is associated with significant mortality and morbidity in mothers and offspring.^2^, ^3^ No definite cure other than delivery is known.^4^

Offspring exposed to maternal preeclampsia have higher blood pressure and body mass index (BMI) during childhood and early adolescence.^5^, ^6^ Exposure to maternal preeclampsia has also been reported to be associated with a greater risk of stroke in adulthood.^7^ Thus, the worse cardiovascular disease (CVD) risk profile observed in exposed offspring in adolescence might translate into CVD events later in life. To facilitate risk stratification and preventive efforts, it is important to identify potential CVD risk factors and pathways to disease. Noticeably, both preeclampsia and gestational hypertension are associated with greater long‐term CVD risk in exposed mothers.^8^, ^9^ Shared genetic risk between the mother and offspring may thus be a contributor of increased offspring risk. However, offspring exposed to maternal preeclampsia have decreased flow‐mediated dilation, which was not observed in their unexposed siblings, suggesting a direct intrauterine effect.^10^ Furthermore, a direct influence of exposure to preeclampsia on fetal cardiac development is plausible,^11^ given that altered pressure loads and hypoxia during development of the myocardium might have a long‐lasting effect on its structure and function.^12^, ^13^ The increase in number of myocytes in the human myocardium plateaus during early life, and subsequent cardiac growth is mostly mediated through hypertrophy of previously formed myocytes.^14^

Our aim was to investigate whether maternal preeclampsia and gestational hypertension were associated with offspring cardiac structure and function in adolescence. As recently demonstrated using data from the same cohort,^15^ patterns of maternal blood pressure (BP) change in pregnancy are associated with birth weight and gestational length. Therefore, we also examined associations between rate of maternal BP change in pregnancy and offspring cardiac outcomes. We hypothesized that (1) cardiac outcomes would be less favorable in offspring exposed to maternal preeclampsia and gestational hypertension, compared to offspring of normotensive mothers, and (2) adverse maternal BP change during pregnancy would be associated with poorer offspring cardiac structure and function.

## Methods

**Figure 1:**
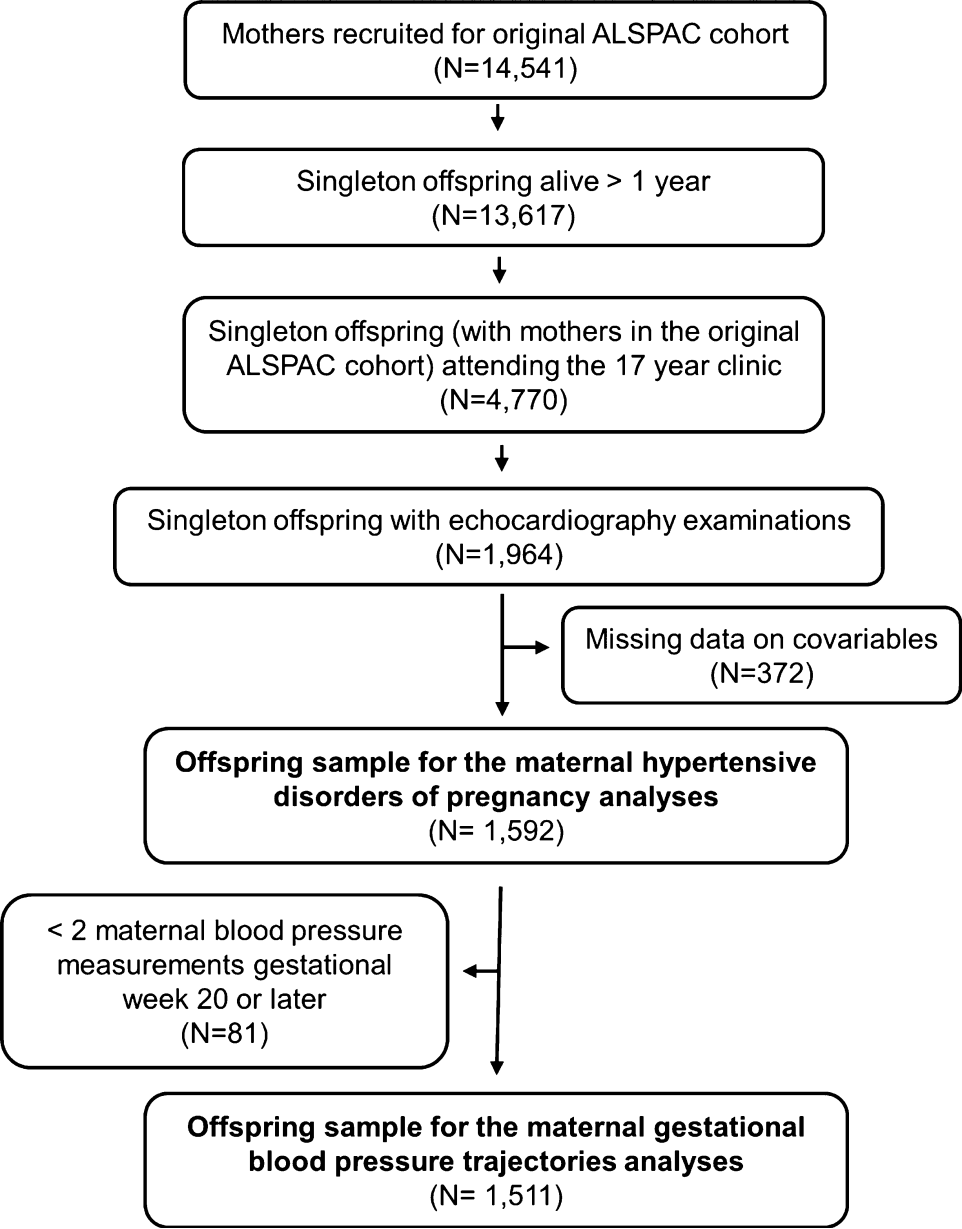
Study sample flow chart. ALSPAC indicates Avon Longitudinal Study of Parents and Children.

The Avon Longitudinal Study of Parents and Children (ALSPAC) is a prospective, population‐based birth cohort study that recruited 14 541 pregnant women resident in the county of Avon, United Kingdom, with expected dates of delivery between April 1, 1991 and December 31, 1992. There were 13 617 mother‐offspring pairs from singleton live births who survived to ≥1 year of age. Of these, 4770 offspring attended the follow‐up clinic at age 17 years when a random subsample also underwent echocardiography (N=1964 singletons from the original cohort). The current study sample includes all singleton offspring with complete data on all covariables (N=1592; 81.1% of singletons who underwent echocardiography; see Figure 1). Please note that the study website contains details of all the data that are available through a fully searchable data dictionary (http://www.bris.ac.uk/alspac/researchers/data-access/data-dictionary/). Ethical approval for the study was obtained from the ALSPAC Ethics and Law Committee and the local research ethics committee, and informed consent was obtained from all participants. The study complies with the Declaration of Helsinki.

### Maternal Data

Data collection in the ALSPAC mothers has been previously described in detail.^16^ Single brachial BP was measured in the seated position using the appropriate cuff size at regular antenatal care visits. All of the BP and urine dipstick proteinuria and glycosuria measurements, which were taken routinely as part of antenatal care, were abstracted from obstetric medical charts by 6 trained research midwives. There was no between‐midwife variation in mean values of the data abstracted, and error rates were consistently <1% in repeated data entry checks. Using these data, we classified the pregnant women into 1 of 4 mutually exclusive categories of hypertension during pregnancy: normotensive, preeclampsia, gestational hypertension, or essential hypertension. We defined gestational hypertension as hypertension manifesting during pregnancy according to the criteria of the International Society for the Study of Hypertension in Pregnancy^17^ (systolic BP [SBP] ≥140 mm Hg or diastolic BP [DBP] ≥90 mm Hg on at least 2 occasions after the 20th gestational week). For the definition of preeclampsia, we used the BP limits as above but with concomitant proteinuria, measured with a dipstick, on both hypertensive occasions. In addition, we categorized those exposed to maternal preeclampsia superimposed on essential hypertension in the preeclampsia group. Women who reported having been diagnosed previously with hypertension outside of pregnancy and were aged >16 years at diagnosis were considered to have essential hypertension. We included offspring born to these mothers with essential hypertension during pregnancy as a separate exposure group for comparison and for completeness. We also identified women with glycosuria or diabetes mellitus based on antenatal care records. Glycosuria was defined as a record of at least ++ (equal to 13.9 mmol/L or 250 mg/100 mL) on at least 2 occasions at any time during the pregnancy. Information on gestational length was also abstracted from medical records. Information on prepregnancy weight, height, maternal level of education, and maternal smoking during pregnancy were collected by questionnaires during early pregnancy. Maternal smoking was categorized as never, stopped before the second trimester, or smoked throughout pregnancy. Maternal level of education was categorized as compulsory/vocational, compulsory with higher academic achievement, secondary with academic preparation, or university degree.

### Offspring Data

During the age 17 clinic assessment, offspring brachial BP (typically from the right arm) was measured with the subject in a sitting position using an Omron 705‐IT machine (Omron Healthcare Ltd, Milton Keynes, UK). The mean of 2 readings, respectively, was used in this study. Mean arterial pressure (MAP) was calculated using SBP and DBP as MAP=DBP+(SBP−DBP)/3. Weight was measured in light clothing to the nearest 0.1 kg by using Tanita scales (Tanita Europe BV, Amsterdam, The Netherlands). Height was measured without shoes to the nearest 0.01 m using a Harpenden stadiometer (Holtain Ltd, Crymych, UK). BMI was calculated as (weight in kilograms)/(height in meters)^2^. Offspring birth weight was extracted from obstetric records as described for the mothers above.

#### Echocardiography examinations in offspring

All measurements were performed according to American Society of Echocardiography guidelines. Echocardiography was performed by 1 of 2 experienced echocardiographers following a standard examination protocol using an HDI 5000 ultrasound machine (Phillips Healthcare, Amsterdam, The Netherlands) equipped with a P4‐2 Phased Array ultrasound transducer. As outcomes, we focused on 3 measures of cardiac structure (relative wall thickness, left ventricular mass indexed to height in m^2.7^ [LVMI], and left ventricular end diastolic volume), systolic function (ejection fraction [EF], midwall fractional shortening [MFS], and left ventricular wall velocity as measured with tissue doppler [s′]), and diastolic function (E/A wave ratio, E/e′ wave ratio, and left atrial diameter indexed to height in m^2.7^ [LADI]), respectively. Though LADI is not a strictly functional measure, the size of the left atrium is an indicator of left ventricular diastolic function. Mean fractional shortening (MFS) and left atrial diameter were measured using M‐mode. Tissue Doppler echocardiography was performed in the 4‐chamber view on the lateral left ventricular wall to obtain myocardial wall velocities. Peak early diastolic (e′) and atrial contraction (a′) tissue velocities were recorded with a 5‐mm sample volume over the mitral valve annulus for 8 to 10 cycles. Peak systolic left ventricular tissue velocities (s′) was calculated as the average from s′ measurements of the lateral and septal left ventricular wall, both measured as above during 3 time points each. The velocity profile for transmitral flow was recorded with the patient in passive end expiration. Peak flow velocity of the early (E) and atrial (A) waves was measured from the 3 consecutive cardiac cycles displaying the highest measurable velocity profiles. EF was calculated as the percent of the end‐diastolic volume that was ejected each cardiac cycle. Relative wall thickness was calculated using posterior wall thickness in diastole and left ventricular internal diameter in diastole.

Ongoing quality control was performed throughout the study, and reproducibility of echocardiographic examinations was assessed by recalling 30 participants and repeating their measurements. The intraclass correlation of repeated measurements ranged from 0.75 to 0.93 (intraobserver) and 0.78 to 0.93 (interobserver).

### Statistical Analyses

All analyses were conducted in STATA v. 13.1 for Windows (StataCorp LP, College Station, TX). To investigate the association between maternal blood pressure change during pregnancy and offspring cardiac outcomes, we used MLWin through the “runmlwin”^18^ procedure and the “reffadjust” package in STATA. Results are presented with 95% CIs.

#### Preeclampsia, gestational hypertension, and offspring cardiac structure and function

We used linear regression models to study the associations between preeclampsia and hypertension in pregnancy with offspring cardiac outcomes. We considered the following covariables as confounders (occurring before the diagnosis of maternal preeclampsia): maternal age, prepregnancy BMI, parity, glycosuria/diabetes mellitus, educational level, and smoking during pregnancy. Potential mediators were gestational length, offspring birth weight, and offspring BMI and MAP in adolescence.

We used models progressively adjusting for covariables in 3 steps: model I (offspring age and sex); model II (additionally adjusted for maternal age at delivery, parity, glycosuria/diabetes mellitus, smoking during pregnancy, BMI, and educational level); and model III (additionally adjusted for birth weight, gestational length, offspring BMI, and MAP at follow‐up). We consider model II to be our main model because this estimates the direct effect of the exposure on the outcomes of interest.

#### Rate of maternal BP change in pregnancy and offspring cardiac structure and function

To model associations with maternal gestational SBP and DBP change, we constructed BP trajectories using multilevel spline models (measurement occasion by woman) as previously described.^19^, ^20^ First, offspring whose mothers had less than 2 BP measurements following the 20th week of gestation were excluded (N=81). To analyze the maternal BP change during different stages of pregnancy, we modeled DBP and SBP change (each separately) during 4 periods from week 8 onward (gestational weeks 8–18, 18–30, 30–36, and ≥36), each knot point demarcating periods of linear rate of change in BP. To relate maternal BP change to offspring cardiac outcomes, we formed a bivariate model by extending the multilevel models for BP change to include the offspring outcome as an extra response variable at the woman level (level 2). Regression coefficients for the associations of BP at baseline and rates of BP change in each period with the outcomes were derived from the level 2 variance–covariance matrix of the random effects.^15^, ^21^ Here, too, we used progressively adjusted models: model I (offspring age and sex); model II (additionally adjusted for maternal age at delivery, parity, glycosuria/diabetes mellitus, smoking during pregnancy, maternal BMI, maternal educational level, maternal SBP [or DBP] gestational week 8 [baseline], and, when applicable, previous SBP [or DBP] change during pregnancy); model III (additionally adjusted for hypertension during pregnancy); and model IV (additionally adjusted for offspring birth weight, gestational length, offspring BMI at follow‐up, and offspring MAP at follow‐up). Potential confounders that could affect the maternal BP trajectory (offspring sex [model I], maternal age at birth, parity, glycosuria/diabetes mellitus, smoking during pregnancy, maternal BMI, maternal educational level [model II], and maternal hypertension during pregnancy [model III]) were adjusted for by including these in the maternal BP part of the multilevel model. More specifically, these variables were included as main effects and as interactions with each spline to fully adjust for their association with the whole trajectory of maternal BP change. At each stage of adjustment, all confounders and mediators to be adjusted for at that particular stage were also included as covariates in the offspring outcome part of the multilevel model.

## Results

In models of maternal hypertension in pregnancy, 1592 offspring were included whereas 1511 offspring were included in models of BP trajectories (Figure 1). In total, 2.6% of offspring had been exposed to maternal preeclampsia. Table shows the study sample characteristics and Table S1 shows the characteristics in our study sample compared to those who attended the year 17 assessment clinic and to the entire ALSPAC offspring cohort. Offspring examined with echocardiography appeared similar to all offspring who attended the year 17 clinic assessment, including BMI, SBP, and DBP. Mothers of all offspring who attended the year 17 clinic tended to have been somewhat older at delivery, more likely to have had a girl than boy, more likely to have had no previous pregnancies, more likely not to smoke throughout pregnancy, and to have a higher level of education compared to the ALSPAC cohort overall.

**Table 1:** Description of Study Participants by Maternal Hypertensive Status During Pregnancy

	Normotensive	Preeclampsia	Gestational Hypertension	Essential Hypertension	*P* Value for Difference
No. of participants (%)	1260 (79.1)	42 (2.6)	247 (15.5)	43 (2.7)	n/a
Female offspring (%)	697 (55.3)	20 (47.6)	121 (49.0)	19 (44.2)	0.13
Birth weight, kg, mean (SD)	3.44 (0.49)	3.00 (0.80)	3.46 (0.56)	3.34 (0.53)	<0.001
Maternal age at delivery, y, mean (SD)	29.5 (4.5)	30.0 (5.4)	29.2 (4.8)	29.3 (4.7)	0.65
Gestational age at birth, weeks, median (IQR)	40 (39–41)	38.5 (36–40)	40 (39–41)	39 (38–40)	<0.001
Maternal prepregnancy BMI, kg/m^2^, median (IQR)	21.8 (20.4–23.8)	24.1 (21.1–27.5)	23.3 (21.5–26.5)	23.6 (21.8–27.8)	<0.001
First pregnancy (%)	607 (48.2)	30 (71.4)	147 (59.5)	29 (67.4)	<0.001
Diabetes mellitus or glycosuria during pregnancy (%)	36 (2.9)	6 (14.3)	10 (4.0)	2 (4.7)	<0.001
Maternal smoking status during pregnancy (%)					0.03
Never smoked	998 (79.2)	33 (78.6)	210 (85.0)	34 (79.1)	
Stopped before the second trimester	100 (7.9)	7 (16.7)	21 (8.5)	3 (7.0)	
Smoked during the second trimester	162 (12.9)	2 (4.8)	16 (6.5)	6 (14.0)	
Maternal educational level (%)					0.02
Compulsory/vocational	238 (18.9)	10 (23.8)	39 (15.8)	4 (9.3)	
Compulsory (higher achievement)	405 (32.1)	13 (31.0)	100 (40.5)	23 (53.5)	
Secondary (academic preparation)	351 (27.9)	14 (33.3)	54 (21.9)	8 (18.6)	
Tertiary/degree	266 (21.1)	5 (11.9)	54 (21.9)	8 (18.6)	
Measures from the 17 years follow‐up clinic					
Offspring age, y, mean	17.7 (0.3)	17.7 (0.3)	17.7 (0.3)	17.6 (0.3)	0.35
Offspring BMI, kg/m^2^, median (IQR)	21.7 (19.9–24.4)	22.7 (19.9–24.8)	22.8 (20.7–25.7)	22.4 (19.4–24.7)	<0.001
Offspring MAP, mm Hg, mean (SD)	81.5 (6.7)	84.4 (6.2)	83.6 (7.0)	83.3 (6.7)	<0.001
Cardiac structure					
Relative wall thickness, mean (SD), N=1591	0.37 (0.05)	0.40 (0.06)	0.38 (0.6)	0.39 (0.07)	0.008
LVMI, g/m^2.7^, mean (SD)	29 (6)	29 (7)	30 (7)	31 (7)	0.003
LVED volume, mL, mean (SD)	94 (21)	89 (21)	97 (23)	97 (21)	0.06
Systolic function					
Ejection fraction, %, mean (SD)	67 (6)	68 (5)	67 (6)	68 (5)	0.19
Midwall fractional shortening, %, mean (SD), N=1591	16 (2)	16 (2)	16 (2)	16 (2)	0.97
Average s′, cm/s, mean (SD), N=1522	7.8 (1.5)	8.3 (1.7)	7.9 (1.4)	7.5 (1.2)	0.11
Diastolic function					
Mitral E/A ratio, mean (SD), N=1533	1.9 (0.4)	1.9 (0.5)	1.9 (0.4)	2.0 (0.4)	0.19
Lateral E/e′ ratio mean (SD), N=1520	4.9 (1.0)	4.8 (0.9)	4.8 (1.0)	5.0 (1.2)	0.67
LADI, cm/m^2.7^, mean (SD), N=1426	0.75 (0.12)	0.77 (0.13)	0.75 (0.13)	0.74 (0.14)	0.78

BMI indicates body mass index; IQR, interquartile range; LADI, left atrial diameter indexed to height in m^2.7^; LVED volume, left ventricular end‐diastolic volume; LVMI, left ventricular mass indexed to height in m^2.7^; MAP, mean arterial pressure; n/a, not applicable.

**Figure 2:**
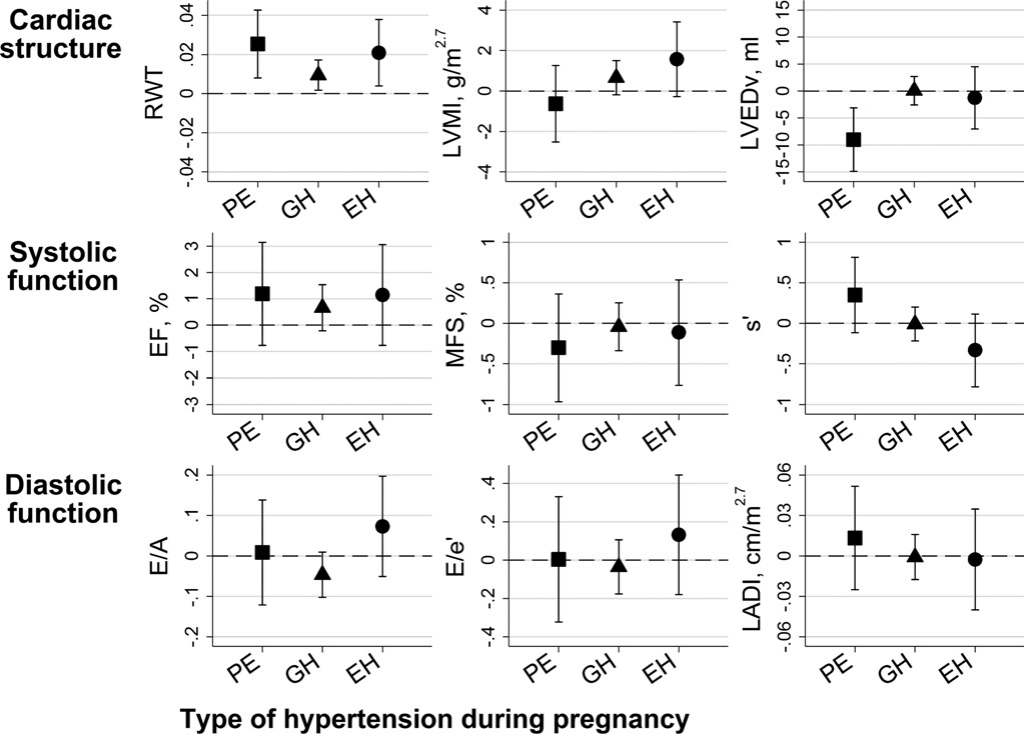
Mean difference (95% CI) in adolescent echocardiography measures between offspring exposed to preeclampsia, gestational hypertension, and essential hypertension in utero compared to offspring of normotensive women. Results obtained from the main model (model II). EF indicates ejection fraction; EH, essential hypertension; GH, gestational hypertension; LADI, left atrial diameter indexed to height in m^2.7^; LVEDv, left ventricular end diastolic volume; LVMI, left ventricular mass indexed to height in m^2.7^; MFS, left ventricular midwall fractional shortening; PE, preeclampsia; RWT, relative wall thickness.

Associations between maternal preeclampsia, gestational hypertension, and essential hypertension and offspring cardiac structure and function from our main confounder adjusted model (model II) are summarized in Figure 2 (Table S2 shows results from all models). Mean RWT was greater in offspring exposed to a hypertensive disorder during pregnancy, whereas only offspring exposed to maternal preeclampsia had smaller mean LVED volume. We observed similar results in a sensitivity analyses using LVED volume indexed to height in m^2.7^ (data not shown). Maternal preeclampsia and essential hypertension were associated with greater offspring RWT in all 3 models. Gestational hypertension was also associated with greater RWT in model II. Gestational hypertension and essential hypertension were positively associated with LVMI in model I, but the association persisted only for essential hypertension after further adjustment for maternal and offspring factors. We found no association between hypertension in pregnancy and offspring cardiac function.

**Figure 3:**
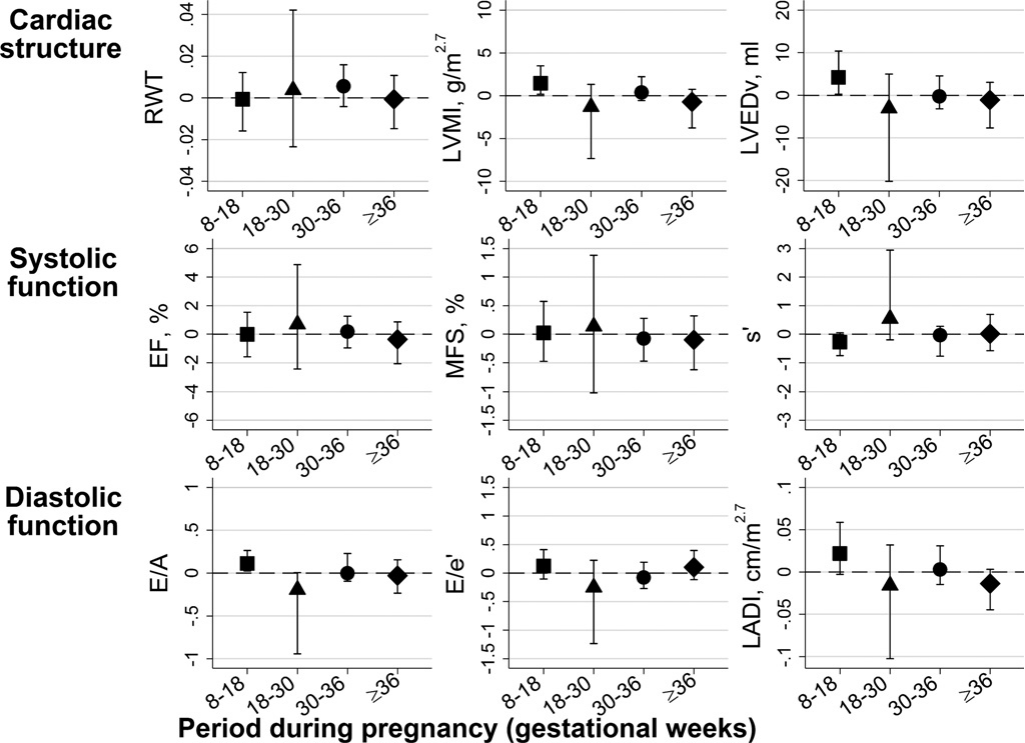
Mean difference (95% CI) in adolescent offspring echocardiography measures per mm Hg/week rate of maternal systolic blood pressure change during pregnancy. Results obtained from the main model (model II). EF indicates ejection fraction; EH, essential hypertension; MFS, left ventricular midwall fractional shortening; LADI, left atrial diameter indexed to height in m^2.7^; LVEDv, left ventricular end‐diastolic volume; LVMI, left ventricular mass indexed to height in m^2.7^; RWT, relative wall thickness.

Figure 3 shows associations between rate of maternal SBP change across pregnancy with measures of offspring cardiac structure and function from model II (main confounder adjusted model). Rate of maternal SBP change during weeks 8 to 18 was positively associated with offspring LVMI, LVED volume, and E/A (ie, a shallower decrease in maternal SBP was associated with offspring outcomes). Full results from all models are presented in Table S3.

**Figure 4:**
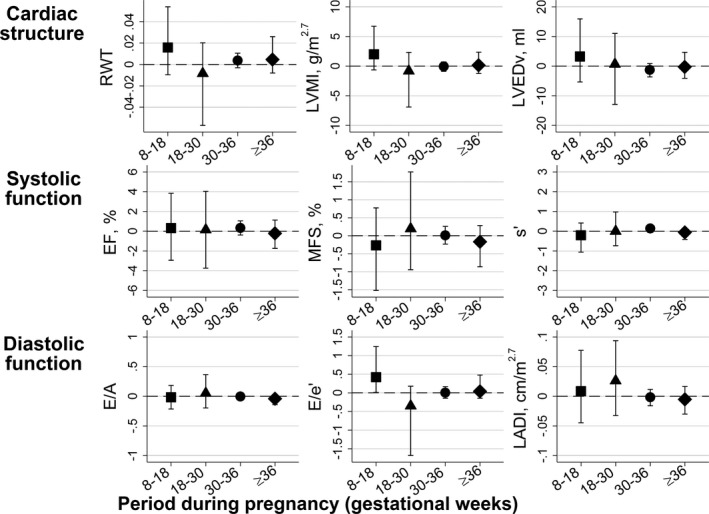
Mean difference (95% CI) in adolescent offspring echocardiography measures per mm Hg/week rate of maternal diastolic blood pressure change during pregnancy. Results obtained from the main model (model II). EF indicates ejection fraction; EH, essential hypertension; MFS, left ventricular midwall fractional shortening; LADI, left atrial diameter indexed to height in m^2.7^; LVEDv, left ventricular end‐diastolic volume; LVMI, left ventricular mass indexed to height in m^2.7^; RWT, relative wall thickness.

Rate of maternal DBP change during weeks 8 to 18 was positively associated with E/e′ (Figure 4). No other association was found between rate of maternal DBP change during pregnancy and offspring cardiac outcomes (Table S4).

## Discussion

In this cohort study, we found offspring exposed to maternal preeclampsia to have a distinct cardiac structure approaching concentric remodeling. Compared to offspring of normotensive women, they had greater mean RWT and lower mean LVED volume, but no difference in LVMI. Offspring exposed to maternal gestational hypertension had increased RWT without any concomitant decrease in LVED volume, and we observed similar results in offspring of mothers with essential hypertension in our main model. However, we found no associations between maternal preeclampsia and offspring cardiac function and no consistent association between rate of maternal BP change throughout pregnancy and offspring cardiac outcomes. The few associations between maternal BP changes and offspring cardiac outcomes that we did observe, and which were not attenuated when adjusted for potential confounders or mediators, appertained to early pregnancy. More specifically, we found maternal SBP change during gestational weeks 8 to 18 to be associated with offspring cardiac mass (LVMI), LVED volume, and a measure of diastolic function (E/A).

Several, but not all, studies have suggested that exposure to maternal preeclampsia or hypertension during pregnancy is associated with adverse cardiac or vascular structure and function in offspring in childhood^10^, ^22^, ^23^, ^24^ and later in life.^25^ Fugelseth et al^22^ found that children exposed to preeclampsia (N=25) have smaller hearts compared to nonexposed at age 5 to 8 years, but the reference group included a large proportion of diabetic pregnancies. Similar to our results, Himmelmann et al^23^ observed no difference in left ventricular mass when comparing offspring born following hypertensive pregnancies to unexposed offspring (N=52; age, 10–16 years). In this study, we found no evidence of suboptimal cardiac function in adolescent offspring exposed to preeclampsia whereas Lewandowski et al^25^ reported a decrease in the global longitudinal peak systolic strain in adult offspring exposed to maternal preeclampsia (N=29). However, these offspring were born preterm and were also older (20–39 years) than our sample, which limits comparison. Importantly, left ventricular concentric remodeling is associated with increased risk of coronary heart disease and stroke in middle‐aged and older adults.^26^ Given that concentric remodeling increases with age,^27^ the suboptimal cardiac structure we observed in offspring exposed to maternal preeclampsia might further worsen with age and exposure to additional CVD risk factors across the life course.

Women with preeclampsia have altered left ventricular geometry compared to those with a normal pregnancy,^28^ which only partly is reversible postpartum.^29^ Likewise, women with gestational hypertension have higher RWT, a measure of left ventricular concentricity, in late pregnancy compared to normotensive pregnant women,^30^ and RWT in adolescence seems to have a substantial heritable component (≈30%).^31^ Thus, women with altered cardiac geometry or greater RWT might be both more likely to have preeclampsia or hypertension during pregnancy, as well as to have offspring with altered cardiac geometry and higher RWT simply because of shared genes between mother and offspring. An alternative explanation is that exposure to maternal hypertension in utero, or the processes causing the hypertension, has a direct effect on offspring cardiac development resulting in altered structure in adolescence.

High resistance in the umbilical artery has been shown to be associated with low cardiac mass in preschool offspring,^32^ suggesting an association between the hemodynamics in utero and later cardiac structure and function. Furthermore, Kuznetsova et al reported that offspring measures of cardiac structure, such as left ventricular mass, have a higher correlation with maternal than paternal measurements,^33^ lending some support for a direct maternal‐specific effect.

Higher BP is a risk factor for cardiac remodeling and preeclampsia is a risk factor for small birth size,^34^ which is associated with more‐adverse cardiovascular development in the offspring.^35^ When accounting for offspring factors (birth weight, gestational length, BMI, and MAP) in model III, the association between exposure to maternal preeclampsia or essential hypertension and later RWT remained, but was abolished for maternal gestational hypertension. The association between maternal preeclampsia and offspring LVED volume was similar in models II and III. Thus, the association between maternal preeclampsia and offspring cardiac structure measures seems to be independent of offspring's own BP or birth size. That said, this approach assumes no measurement error in the mediators and this cannot be ruled out. We found that women with smaller decreases in SBP in weeks 8 to 18 had offspring with larger LVMI, LVED volume, and less optimal diastolic function. A previous study on mothers in the ALSPAC cohort also showed that those with gestational hypertension had a smaller decrease in BP than normotensive mothers during gestational weeks 8 to 18 (Figure S1).^19^ Early pregnancy is a sensitive period for fetal cardiac development, and it is possible that maternal BP, or other cardiovascular adaptions to pregnancy, has a direct effect on the development of offspring cardiac structures. Cardiac structures develop early in the human fetus, and by the end of the first trimester they are largely developed and functional.^36^ The implications of these associations for offspring cardiac development are not yet known.

The main strengths of this study are the prospective cohort design, the available data on multiple possible confounders and mediators, and the number of repeated measures of maternal BP during pregnancy. The study data were mostly collected as part of routine antenatal care in the UK, a structured and low‐threshold health care setting. Maternal BP and the concomitant urine dipstick measurements were collected as part of routine maternity visits, though there was little standardization in terms of the frequency of visits or the time of day. However, few cases should have been missed in the sample, and the use of routine measurements meant that we were able to apply standard definitions of preeclampsia and gestational hypertension to all women rather than relying on recorded diagnoses. The study has some potential limitations: Late adolescence might be too early to detect changes in cardiac function that progress with age. The study sample might have differed somewhat from the total ALSPAC cohort, with offspring of older and more‐educated women being more likely to remain engaged with the study. That said, we cannot think of a plausible reason why this would induce major bias in the association between preeclampsia and the echocardiography outcomes. Despite the large sample size, the number of participants exposed to preeclampsia was limited. Another limitation is that we do not have data on any medical action taken based on a maternal diagnosis of preeclampsia or hypertension. Finally, we conducted multiple tests, and though these were not all independent of one another, it is possible that some reported associations could be attributed to chance. Therefore, focus should be on the pattern of reported observations, and caution is required in the interpretation of specific associations until these are further replicated.

## Conclusion

Adolescent offspring exposed in utero to maternal preeclampsia had evidence of early unfavorable changes in cardiac structure with increased mean RWT and reduced LVED volume (ie, a concentric type of remodeling). Offspring exposed to gestational hypertension had higher mean RWT only. Our results support the hypothesis that fetal exposure to preeclampsia is associated with a more‐adverse cardiac structure in youth. The long‐term implications of these findings for cardiovascular health remain to be established, and further replication of specific associations is warranted.

## Disclosures

None.

## Supporting information


**Table S1.** Description of Study Sample
**Table S2.** Association Between Preeclampsia or Hypertension During Pregnancy and Adolescent Offspring Cardiac Structure and Function Shown as Mean Differences Compared to Offspring of Normotensive Women
**Table S3.** Association Between Rate of Maternal Systolic Blood Pressure Change During Pregnancy and Offspring Cardiac Outcomes in Adolescence
**Table S4.** Association Between Rate of Maternal Diastolic Blood Pressure Change During Pregnancy and Offspring Cardiac Outcomes in Adolescence
**Figure S1.** Description of the maternal blood pressure trajectories during pregnancy by hypertensive disorders of pregnancy in the ALSPAC cohort. Reproduced with permission from *Hypertension*.^1^
