# Supplementary material for: Hypertensive Disorders of Pregnancy and Offspring Cardiac Structure and Function in Adolescence
Source: J Am Heart Assoc. 2016 Oct 31;5(11):e003906. doi: 10.1161/JAHA.116.003906 (PMC5210338; doi:10.1161/JAHA.116.003906)
Supplement: Supplementary file 1 — Table S1. Description of Study Sample Table S2. Association Between Preeclampsia or Hypertension During Pregnancy and Adolescent Offspring Cardiac Structure and Function Shown as Mean Differences Compared to Offspring of Normotensive Women Table S3. Association Between Rate of Maternal Systolic Blood Pressure Change During Pregnancy and Offspring Cardiac Outcomes in Adolescence Table S4. Association Between Rate of Maternal Diastolic Blood Pressure Change During Pregnancy and Offspring Cardiac Outcomes in Adolescence Figure S1. Description of the maternal blood pressure trajectories during pregnancy by hypertensive disorders of pregnancy in the ALSPAC cohort. Reproduced with permission from Hypertension.1 [file JAH3-5-e003906-s001.pdf]

SUPPLEMENTAL MATERIAL

Table S1. Description of study sample

Obstetric and maternal variables	Study sample	Total follow-up, age 17 year clinic	ALSPAC offspring alive > age 1 years
Number of subjects (singletons with birth data)	1,592	4,770	13,617
Female offspring, N (%)	857 (53.8)	2,676 (56.1)	6,594 (48.4)
Birth weight, kg Mean (SD)	3.43 (0.51)	3.43 (0.52, N=4,717)	3.41 (0.54, N=13,446)
Maternal age at delivery, years Mean (SD)	29.5 (4.6)	29.2 (4.7)	28.0 (5.0)
Gestational length, weeks Median (IQR)	40 (39-41)	40 (39-41)	40 (39-41)
Maternal pre-pregnancy BMI, Kg/m^2 Median (IQR)	22.2 (20.5–24.4)	22.1 (20.5–24.2, N=4,330)	22.9 (20.5-24.7, N=11,255)
First pregnancy, N (%)	813 (51.1)	2,223 of 4,612 (48.2)	5,660 of 12,600 (44.9)
Diabetes or glycosuria during pregnancy, N (%)	54 (3.4)	189 of 4,583 (4.1)	494 of 12,047 (4.1)
HDP or hypertension, N (%)		N=4,546	N=13,845
- No HDP or hypertension	1,260 (79.2)	3,613 (79.5)	11,234 (83.8)
- Gestational hypertension	247 (15.5)	673 (14.8)	1,886 (14.4)
- Preeclampsia	42 (2.6)	107 (2.4)	281 (2.1)
- Essential hypertension	43 (2.7)	153 (3.4)	444 (3.2)
Maternal smoking status during pregnancy, N (%)		N=4,674	N=12,777
- Never smoked	1,275 (80.1)	3,595 (76.9)	9,520 (74.5)
- Stopped prior the second trimester	131 (8.2)	486 (10.4)	885 (6.9)
- Smoked during the second trimester	186 (11.7)	593 (12.7)	2,372 (18.6)
Maternal educational level, N (%)		N=4,604	N=12,102
- Compulsory/Vocational	291 (18.3)	896 (19.5)	2,439 (20.2)
- Compulsory (higher achievement)	541 (34.0)	1,571 (34.1)	1,196 (30.0)
- Secondary (academic preparation)	427 (26.8)	1,277 (27.7)	4,185 (34.6)
- Tertiary/Degree	333 (20.9)	860 (18.7)	1,554 (12.8)
**Measures from the 17 years follow-up clinic**			
Offspring age, Mean years (SD)	17.7 (0.3)	17.8 (0.4)	n/a
Offspring BMI, Kg/m^2 Median (IQR)	21.8 (20.0-24.6)	21.9 (20.1-24.6, N=4,632)	n/a
Offspring SBP, mmHg Mean (SD)	118.9 (11.0)	118.5 (10.9, N=4,297)	n/a
Offspring DBP, mmHg Mean (SD)	63.5 (6.5)	63.9 (6.6, N=4,297)	n/a
Offspring MAP, mmHg Mean (SD)	82.0 (6.8)	82.1 (6.8, N=4,297)	n/a

ALSPAC: the Avon Longitudinal Study of Parents and Children, BMI: Body mass index, DBP**:** Diastolic blood pressure, HDP: Hypertension disorders of pregnancy, IQR: Interquartile range, MAP: Mean arterial pressure, SBP: Systolic blood pressure, SD: Standard deviation

Table S2. The association between preeclampsia or hypertension during pregnancy and adolescent offspring cardiac structure and function shown as mean differences compared to offspring of normotensive women.

**Outcome**	**Model**	**Preeclampsia**			**Gestational hypertension**			**Essential hypertension**		
		Mean	95 % CI		Mean	95 % CI		Mean	95 % CI	
Cardiac structure										
RWT	I*	**0.021**	0.004	0.038	0.007	-0.0005	0.015	**0.017**	0.001	0.034
	II†	**0.025**	0.008	0.043	**0.010**	0.002	0.017	**0.021**	0.004	0.038
	III‡	**0.021**	0.004	0.039	0.007	-0.0003	0.015	**0.019**	0.002	0.036
LVMI, g/$m^{2.7}$	I	0.001	-1.9	1.9	**1.2**	0.33	2.0	**2.2**	0.31	4.0
	II	-0.63	-2.5	1.3	0.66	-0.19	1.5	1.6	-0.27	3.4
	III	-0.52	-2.2	1.1	0.15	-0.57	0.88	**1.8**	0.19	3.4
LVEDv, ml	I	**-6.7**	-13	-0.8	1.7	-0.9	4.3	0.9	-4.9	6.6
	II	**-9.0**	-15	-3.1	0.1	-2.6	2.7	-1.3	-7.0	4.5
	III	**-6.7**	-12	-1.1	-0.6	-3.1	1.9	-0.4	-5.9	5.1
Systolic function										
EF, %	I	1.6	-0.34	3.5	0.81	-0.04	1.7	1.2	-0.66	3.1
	II	1.2	-0.77	3.1	0.66	-0.21	1.5	1.1	-0.77	3.1
	III	1.0	-1.0	3.0	0.64	-0.24	1.5	1.1	-0.84	3.0
MFS, %	I	-0.10	-0.75	0.56	0.05	-0.24	0.34	-0.01	-0.65	0.63
	II	-0.30	-0.96	0.36	-0.04	-0.34	0.25	-0.11	-0.76	0.54
	III	-0.26	-0.93	0.42	0.0003	-0.30	0.30	-0.09	-0.74	0.56
s'	I	0.43	-0.03	0.89	0.05	-0.16	0.25	-0.27	-0.71	0.18
	II	0.35	-0.12	0.82	-0.01	-0.22	0.20	-0.33	-0.78	0.11
	III	0.30	-0.18	0.77	-0.01	-0.22	0.20	-0.36	-0.81	0.09
Diastolic function										
E/A	I	-0.01	-0.13	0.12	-0.05	-0.11	0.003	0.07	-0.06	0.19
	II	0.01	-0.12	0.14	-0.05	-0.10	0.010	0.07	-0.05	0.20
	III	0.02	-0.11	0.15	-0.04	-0.09	0.020	0.08	-0.04	0.20
E/e'	I	-0.042	-0.36	0.28	-0.052	-0.19	0.09	0.10	-0.20	0.41
	II	0.004	-0.32	0.33	-0.035	-0.18	0.11	0.13	-0.18	0.44
	III	-0.003	-0.33	0.33	-0.021	-0.16	0.12	0.13	-0.18	0.43
LADI, cm/$m^{2.7}$	I	0.024	-0.014	0.062	0.005	-0.011	0.022	0.006	-0.032	0.043
	II	0.013	-0.025	0.052	-0.001	-0.018	0.016	-0.003	-0.040	0.035
	III	0.011	-0.025	0.046	-0.008	-0.023	0.008	0.004	-0.031	0.038

95 % CI: 95 % confidence interval, A: Velocity of the late filling wave of the left ventricle in diastole, e': Tissue velocity of the lateral mitral annulus during early diastolic filling of the left ventricle, E: Velocity of early filling wave of the left ventricle in diastole, EF: Ejection Fraction, EH: Essential hypertension, MFS: Midwall fractional shortening, LADI: Left atrial diameter indexed to height in $m^{2.7}$, LVEDv: Left ventricular end diastolic volume, LVMI: Left ventricular mass indexed to height in $m^{2.7}$, RWT: Relative wall thickness, s': Average tissue velocity of the septal and lateral left ventricular wall in systole

* Model I adjusted for offspring sex, offspring age at follow-up

† Model II additionally adjusted for maternal age, diabetes/glycosuria during pregnancy, parity, smoking during pregnancy, maternal body mass index, maternal education

‡ Model III additionally adjusted for offspring birth weight, gestational length, offspring body mass index, offspring mean arterial pressure

Table S3. The association between rate of maternal systolic blood pressure change during pregnancy and offspring cardiac outcomes in adolescence

**Outcome**	**Model**	**Rate of blood pressure change during gestation** (mmHg/week)											
		Week 8-18			Week 18-30			Week 30-36			≥ Week 36		
		Mean	95 % CI		Mean	95 % CI		Mean	95 % CI		Mean	95 % CI	
Cardiac structure													
RWT	I*	-0.005	-0.020	0.007	0.003	-0.019	0.028	0.005	-0.002	0.012	-0.001	-0.009	0.006
	II†	-0.001	-0.016	0.012	0.004	-0.023	0.042	0.006	-0.004	0.016	-0.001	-0.015	0.011
	III‡	-0.003	-0.017	0.010	0.003	-0.026	0.044	0.004	-0.008	0.015	-0.001	-0.017	0.013
LVMI,	I	**1.4**	0.14	3.5	-1.7	-5.2	0.6	0.19	-0.5	1.0	-0.53	-1.5	0.2
g/m$^{2.7}$	II	**1.5**	0.17	3.5	-1.3	-7.3	1.3	0.37	-0.6	2.2	-0.73	-3.8	0.8
	III	**1.1**	0.03	2.9	-1.5	-7.7	0.9	-0.05	-1.1	1.8	-0.82	-4.1	0.9
LVEDv,	I	**4.9**	0.89	11	-4.5	-15	2.6	-0.52	-2.9	1.8	-0.6	-3.2	1.8
ml	II	**4.2**	0.27	10	-3.1	-20	5.0	-0.25	-3.2	4.5	-1.1	-7.7	3.0
	III	3.6	-0.10	9.3	-3.5	-22	4.5	0.25	-3.2	5.9	-1.7	-11	3.2
Systolic function													
EF, %	I	0.02	-1.5	1.6	0.29	-2.3	3.1	0.19	-0.56	1.0	-0.20	-1.1	0.55
	II	-0.01	-1.6	1.5	0.70	-2.4	4.9	0.18	-0.96	1.3	-0.36	-2.1	0.87
	III	-0.07	-1.6	1.5	0.53	-2.9	4.9	-0.03	-1.4	1.2	-0.48	-2.6	0.97
MFS, %	I	0.15	-0.34	0.70	0.04	-0.9	1.0	-0.06	-0.33	0.19	-0.04	-0.33	0.22
	II	0.02	-0.48	0.57	0.14	-1.0	1.4	-0.08	-0.47	0.28	-0.10	-0.62	0.32
	III	0.05	-0.44	0.60	0.13	-1.2	1.4	-0.09	-0.56	0.34	-0.13	-0.81	0.36
s'	I	-0.26	-0.73	0.08	0.47	-0.11	1.5	0.0002	-0.18	0.19	-0.004	-0.18	0.17
	II	-0.28	-0.75	0.05	0.54	-0.20	2.9	-0.038	-0.78	0.27	0.015	-0.58	0.69
	III	-0.30	-0.77	0.03	0.58	-0.24	3.6	-0.053	-1.0	0.38	0.030	-0.69	1.0
Diastolic function													
E/A	I	**0.11**	0.02	0.26	**-0.16**	-0.43	-0.01	-0.003	-0.05	0.05	-0.02	-0.07	0.02
	II	**0.11**	0.02	0.26	-0.19	-0.94	0.01	-0.002	-0.10	0.23	-0.03	-0.23	0.16
	III	**0.13**	0.04	0.28	-0.18	-1.1	0.03	-0.007	-0.14	0.25	-0.03	-0.27	0.19
E/e'	I	0.11	-0.12	0.40	-0.22	-0.75	0.17	-0.072	-0.20	0.05	**0.11**	0.01	0.25
	II	0.13	-0.10	0.41	-0.25	-1.2	0.22	-0.076	-0.27	0.19	0.10	-0.11	0.39
	III	0.13	-0.10	0.41	-0.22	-1.3	0.30	-0.12	-0.36	0.18	0.11	-0.13	0.46
LADI,	I	0.023	-0.003	0.059	-0.02	-0.08	0.02	0.0003	-0.015	0.015	**-0.012**	-0.029	-0.0004
cm/m$^{2.7}$	II	0.022	-0.003	0.059	-0.02	-0.10	0.03	0.0029	-0.015	0.031	-0.014	-0.045	0.0032
	III	0.022	-0.0003	0.058	-0.01	-0.10	0.04	-0.010	-0.035	0.015	-0.010	-0.038	0.013

95 % CI: 95 % confidence interval, A: Velocity of the late filling wave of the left ventricle in diastole, e': Tissue velocity of the lateral mitral annulus during early diastolic filling of the left ventricle, E: Velocity of early filling wave of the left ventricle in diastole, EF: Ejection Fraction. EH: Essential hypertension, MFS: Midwall fractional shortening, LADI: Left atrial diameter indexed to height in m$^{2.7}$, LVEDv: Left ventricular end diastolic volume, LVMI: Left ventricular mass indexed to height in m$^{2.7}$, RWT: Relative wall thickness, s': Average tissue velocity of the septal and lateral left ventricular wall in systole

* Model I adjusted for offspring sex, offspring age at follow-up

† Model II additionally adjusted for maternal age, diabetes/glycosuria during pregnancy, parity, smoking during pregnancy, maternal body mass index, maternal education, maternal systolic blood pressure gestational week 8, and, when applicable, previous rate of blood pressure change during the pregnancy

‡ Model III additionally adjusted for maternal preeclampsia/hypertension during pregnancy, offspring birth weight, gestational length, offspring body mass index, offspring mean arterial pressure

Table S4. The association between rate of maternal diastolic blood pressure change during pregnancy and offspring cardiac outcomes in adolescence

Outcome	Model	Rate of blood pressure change during gestation (mmHg/week)											
		Week 8-18			Week 18-30			Week 30-36			≥ Week 36		
		Mean	95 % CI		Mean	95 % CI		Mean	95 % CI		Mean	95 % CI	
Cardiac structure													
RWT	I*	0.010	-0.013	0.043	-0.011	-0.042	0.014	0.004	-0.001	0.010	0.002	-0.002	0.006
	II†	0.016	-0.010	0.054	-0.008	-0.057	0.020	0.004	-0.003	0.011	0.005	-0.008	0.026
	III‡	0.012	-0.015	0.050	-0.014	-0.074	0.017	0.001	-0.008	0.009	0.004	-0.019	0.031
LVMI, $g/m^{2.7}$	I	1.9	-0.5	6.4	-1.4	-4.9	1.3	0.17	-0.43	0.79	-0.08	-0.5	0.4
	II	2.0	-0.6	6.7	-0.8	-6.9	2.3	-0.05	-0.89	0.72	0.15	-1.2	2.3
	III	0.7	-1.9	4.0	-0.6	-5.0	2.6	-0.18	-0.94	0.49	-0.36	-2.2	1.4
LVEDv, ml	I	3.8	-3.8	15	-0.7	-10	8.3	-0.85	-2.8	1.0	-0.45	-1.9	1.0
	II	3.2	-5.3	16	0.7	-13	11	-1.3	-3.7	0.9	-0.29	-4.1	4.7
	III	0.7	-9.0	11	2.7	-8.9	16	-0.44	-2.8	1.9	-1.3	-8.8	4.8
Systolic function													
EF, %	I	0.24	-2.5	3.4	-0.05	-3.2	3.0	0.33	-0.28	0.97	-0.16	-0.6	0.3
	II	0.31	-2.9	3.8	0.15	-3.7	4.0	0.32	-0.39	1.0	-0.22	-1.7	1.1
	III	0.34	-2.9	4.3	-0.33	-5.3	3.7	0.16	-0.70	0.99	-0.50	-2.9	1.7
MFS, %	I	-0.15	-1.2	0.8	0.20	-0.8	1.3	0.004	-0.20	0.22	-0.09	-0.25	0.07
	II	-0.27	-1.5	0.8	0.20	-1.0	1.8	0.012	-0.23	0.26	-0.17	-0.86	0.29
	III	-0.16	-1.4	1.0	0.19	-1.2	1.8	0.030	-0.25	0.32	-0.21	-1.2	0.51
s'	I	-0.21	-1.0	0.37	0.047	-0.65	0.76	0.13	-0.006	0.29	-0.037	-0.14	0.07
	II	-0.21	-1.1	0.41	0.001	-0.74	0.97	0.14	-0.024	0.31	-0.064	-0.42	0.12
	III	-0.20	-1.1	0.42	0.025	-0.78	1.1	0.15	-0.034	0.36	-0.060	-0.59	0.24
Diastolic function													
E/A	I	0.007	-0.16	0.19	0.068	-0.14	0.32	-0.010	-0.052	0.031	0.03	-0.06	0.001
	II	-0.016	-0.21	0.18	0.052	-0.20	0.37	-0.004	-0.051	0.044	-0.04	-0.14	0.02
	III	-0.003	-0.20	0.21	0.062	-0.22	0.42	-0.002	-0.055	0.051	-0.05	-0.18	0.05
E/e'	I	**0.43**	0.05	1.3	-0.41	-1.1	0.04	0.023	-0.08	0.13	-0.01	-0.09	0.06
	II	**0.42**	0.01	1.2	-0.35	-1.7	0.18	0.005	-0.14	0.17	0.04	-0.14	0.48
	III	**0.46**	0.05	1.4	-0.43	-2.3	0.15	-0.019	-0.23	0.16	0.05	-0.43	0.68
LADI, $cm/m^{2.7}$	I	0.015	-0.031	0.078	0.01	-0.04	0.07	-0.003	-0.015	0.010	-0.005	-0.014	0.003
	II	0.009	-0.045	0.077	0.03	-0.03	0.09	-0.002	-0.016	0.012	-0.005	-0.030	0.017
	III	-0.003	-0.060	0.054	0.04	-0.02	0.12	-0.008	-0.024	0.007	-0.009	-0.057	0.023

95 % CI: 95 % confidence interval, A: Velocity of the late filling wave of the left ventricle in diastole, e': Tissue velocity of the lateral mitral annulus during early diastolic filling of the left ventricle, E: Velocity of early filling wave of the left ventricle in diastole, EF: Ejection Fraction. EH: Essential hypertension, MFS: Midwall fractional shortening, LADI: Left atrial diameter indexed to height in $m^{2.7}$, LVEDv: Left ventricular end diastolic volume, LVMI: Left ventricular mass indexed to height in $m^{2.7}$, RWT: Relative wall thickness, s': Average tissue velocity of the septal and lateral left ventricular wall in systole

* Model I adjusted for offspring sex, offspring age at follow-up

† Model II additionally adjusted for maternal age, diabetes/glycosuria during pregnancy, parity, smoking during pregnancy, maternal body mass index, maternal education, maternal diastolic blood pressure gestational week 8, and, when applicable, previous rate of blood pressure change during the pregnancy

‡ Model III additionally adjusted for maternal preeclampsia/hypertension during pregnancy, offspring birth weight, gestational length, offspring body mass index, offspring mean arterial pressure

Figure S1.

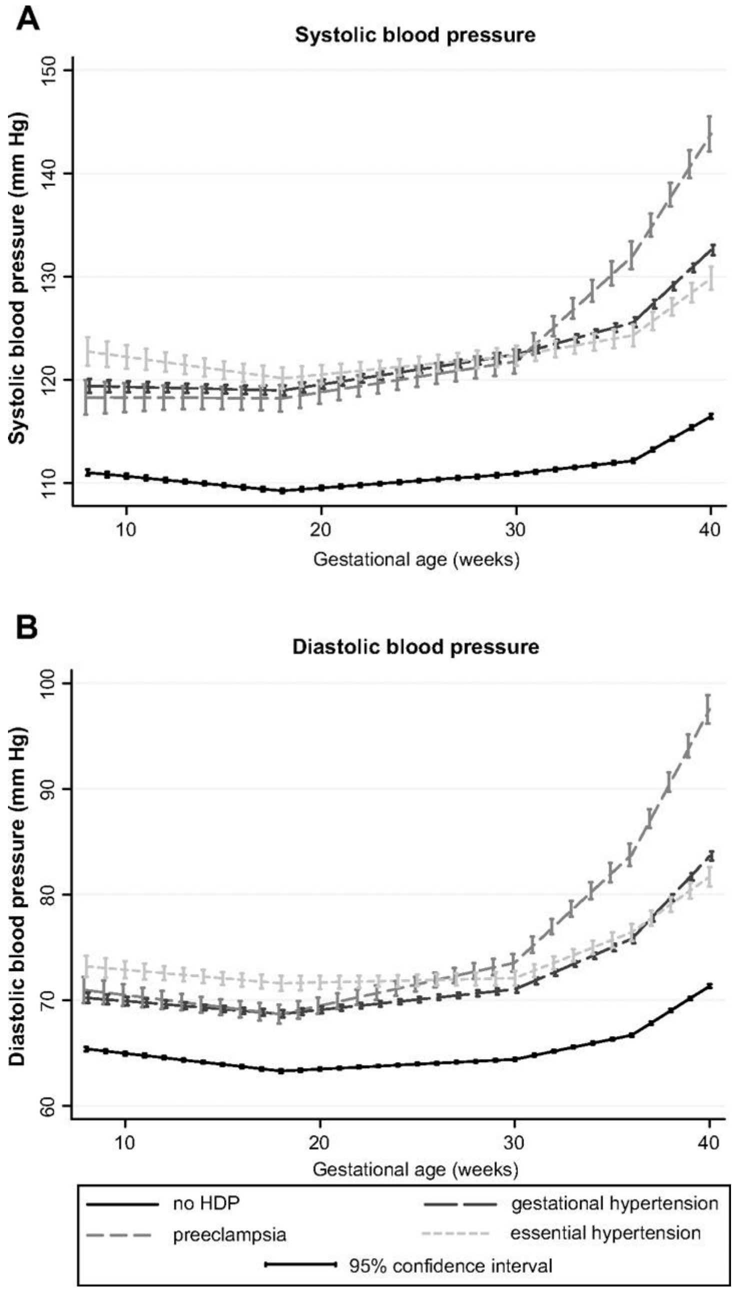

Figure Legend:

Figure S1. Description of the maternal blood pressure trajectories during pregnancy by hypertensive disorders of pregnancy in the ALSPAC cohort. Reproduced with permission from *Hypertension*[1]

Supplemental Reference:

1. Macdonald-Wallis C, Lawlor DA, Fraser A, May M, Nelson SM, Tilling K. Blood pressure change in normotensive, gestational hypertensive, preeclamptic, and essential hypertensive pregnancies. *Hypertension*. 2012;59:1241–1248.
